# Development and Survivability of The Free-Living Stage Larvae of Equine Strongyles in Different Environments and Soil Types

**DOI:** 10.21315/tlsr2025.36.3.5

**Published:** 2025-10-31

**Authors:** Idzana Ab Malek, Fuziaton Baharudin, Tan Li Peng, Jasni Sabri

**Affiliations:** 1 Department of Biomedical Engineering and Health Science, Faculty of Electrical Engineering, Universiti Teknologi Malaysia, 81310 Skudai, Johor, Malaysia; 2 Department of Veterinary Paraclinical Study, Faculty of Veterinary Medicine, Universiti Malaysia Kelantan, 16100 Kota Bharu, Kelantan, Malaysia; 3 Faculty of Veterinary Medicine, Universitas Brawijaya, 65151 Malang, East Java, Indonesia

**Keywords:** Strongyle, Horses, Parasite, Tropical Ambiance, Larval Development, Tropical Soils, Strongylus, Kuda, Parasit, Suhu Tropika, Perkembangan Larva, Tanah Tropika

## Abstract

Equine strongyles are harmful gastrointestinal parasites affecting horses’ health and productivity. In tropical regions, environmental conditions, including temperature and soil type, significantly influence the development and survivability of strongyle larvae. This study aims to assess the development and survival of third-stage larvae (L3) of strongyles under different temperatures and soil types commonly found in Malaysia. Faecal samples from five adult horses aged between 22–24 years that were kept under a semi-intensive management system and had no history of recent anthelmintic treatment for the past six months and did faecal culture at various controlled temperatures: 21 ± 1°C, 26 ± 1°C, 29 ± 1°C and 32 ± 1°C, and soil type (peat, clay, residual and sandy). The presence of strongyle eggs was identified using faecal floatation, while larval development was monitored daily for 30 consecutive days using the Baermann technique and light microscopy. The time required for development from egg to L3 was shortest at 29°C–32°C (5–6 days) and longest at 21°C–26°C (8–9 days). Peat soil supported the highest survivability (up to 68%) compared to sandy and clay soils, which had the lowest survivability (50% and 41%, respectively). This study highlights the role of tropical soil environments in larval survival. It offers insights into improved parasite control strategies, suggesting future investigations using field trials to explore the role of faecal moisture and microbial interactions in L3 persistence.

## Abstract (fr)

**Abstrak:**

Strongylus merupakan parasit gastrointestinal yang memudaratkan kesihatan dan produktiviti kuda. Di kawasan tropika, faktor persekitaran termasuk suhu dan jenis tanah mempengaruhi perkembangan serta kelangsungan hidup larva strongylus. Kajian ini bertujuan menilai perkembangan dan kelangsungan hidup larva peringkat ketiga (L3) strongylus di bawah variasi suhu dan jenis tanah yang biasa ditemui di Malaysia. Sampel najis diambil daripada lima ekor kuda dewasa berumur 22–24 tahun yang dipelihara secara separa intensif dan tidak menerima rawatan anthelmintik dalam tempoh enam bulan sebelumnya. Kultur najis dijalankan pada suhu terkawal: 21 ± 1°C, 26 ± 1°C, 29 ± 1°C dan 32 ± 1°C, serta pada pelbagai jenis tanah (gambut, liat, residu dan berpasir). Kehadiran telur strongylus dikenal pasti menggunakan kaedah apungan najis, manakala perkembangan larva dipantau setiap hari selama 30 hari berturut-turut menggunakan teknik Baermann dan mikroskop cahaya. Tempoh perkembangan daripada telur ke L3 adalah paling singkat pada suhu 29°C –32°C (5–6 hari) dan paling lama pada suhu 21°C–26°C (8–9 hari). Tanah gambut menunjukkan kadar kelangsungan hidup paling tinggi (sehingga 68%) berbanding tanah berpasir dan liat yang mencatatkan kelangsungan hidup terendah (masing-masing 50% dan 41%). Kajian ini menekankan peranan persekitaran tanah tropika terhadap kelangsungan hidup larva serta memberikan pandangan baharu dalam strategi kawalan parasit, di samping mencadangkan kajian lapangan pada masa hadapan untuk meneroka peranan kelembapan najis dan interaksi mikrob dalam kelangsungan L3.

**HIGHLIGHTS**

- Peat soil in moderate ambient temperature at 29 ± 1°C is the most condusive environment for the development and survival rate of equine strongyle larvae in free-living stages.
- High ambient temperature of 32 ± 1°C increased the development rate of the egg to L3 within 5.5 days but reduce the survival rates to 45%.
- In unfavourable soil such as sandy and clay soil; higher ambient temperature reduced the period of larval infectivity in the environment.

## INTRODUCTION

Parasitic infection is a prevalent issue that compromises horses’ health. Equine strongyles (Order: Strongylida) are the most common endoparasites in horses that significantly impact their health and welfare. These nematodes are divided into two main groups: large strongyles (*Strongylus* spp.) and small strongyles (Cyathostominae), both of which inhabit the large intestine of equids. Strongyles have two distinct phases of the life cycle: The development of fourth-stage larvae (L4) into adult worms in the horses’ gastrointestinal tract, where they absorb nutrients from the host, and then expel their eggs into the environment with the horses’ faeces. The second phase involves the hatching of eggs into first-stage larvae (L1), then later developing into second-stage larvae (L2), followed by third-stage larvae (L3). L3 contaminated pastures in the paddock will be ingested by horses while grazing to meet their dietary and nutritional needs (Joó *et al*. 2022).

It is one of the significant causative agents for intestinal necrosis, resulting in colic that may lead to fatality. Studies have shown a correlation between the presence of parasites in the gastrointestinal tract and the occurrence of colic in horses, which is also prevalent across temperate and tropical regions (Kegan & Gary 2018; Hedberg-Alm 2020; Kuzmina *et al*. 2016; Mathewos *et al*. 2021). Strongyle infection in horses can have profound health implications that impact their overall well-being and performance (Emeto *et al*. 2022). The methods for diagnosing strongyle infections in horses include faecal egg counts (Ghafar *et al*. 2021) and monitoring for clinical signs, such as changes in anaemia, appetite, dull coat, lethargy and recurrent colic (Smith *et al*. 2025; Mair & Sherlock 2023). Horse owners must be vigilant and proactive in monitoring their horses’ health and seek veterinary care if they suspect a strongyle infection. Early detection and treatment are crucial in preventing further complications and ensuring the horse’s overall well-being.

Environmental factors such as temperature, humidity and soil type influence the development, survival and dissemination of these larvae (Tzelos *et al*. 2019; Merlin *et al*. 2022). Ambient temperature, humidity, precipitation, soil composition and vegetation structure all have a direct impact on the parasite›s free-living larval stages› growth, survival and transmission (Jas *et al*. 2022). These ecological parameters also play an important role in developing effective parasite control strategies. Optimal larval development occurs at temperatures ranging from 20°C to 30°C and humidity levels exceeding 70%. In contrast, extreme heat or dryness causes larval death (Rose Vineer *et al*. 2020a). Seasonal climatic fluctuations thus influence pasture contamination and the risk of infection.

Despite numerous studies in temperate climates (Kuzmina *et al*. 2016; Bellaw *et al*. 2016; Tydén *et al*. 2019; Joó *et al*. 2022; Tzelos *et al*. 2019), data on tropical regions remain limited (Villa-Mancera *et al*. 2021, Mathewos *et al*. 2021, Molento *et al*. 2024). It is unknown whether the tropical region, such as Malaysia, with year-round high temperatures and humidity, will result in a distinct life cycle of the strongyle. Additionally, the different soil types in this region have different textures, moisture retention, nutrient content and pH levels, which may contribute to a variable environment for the larvae’s survivability, which has not been studied. For example, larvae live longer in moist, loamy soils than in drier, sandy soils (Sánchez *et al*. 2022).

Management of L3 control at the farm is crucial for reducing parasitic infections in horses. With ongoing climate change, strongyle transmission patterns are changing, often expanding risk zones and altering traditional infection peaks (Chylinski *et al*. 2021). Alongside anthelmintic programs, rotational grazing is one of the common parasitic management strategies. Adequate rotational grazing recommends rotating different grazing areas three times a month to break the life cycle of the larvae and reduce the L3 population in various seasonal climates of the temperate region (Donaghy *et al*. 2021). However, the 3-month rotation may not be effective in a tropical environment. Thus, this article emphasises the importance of adaptive and location-specific parasite control strategies, such as selective deworming, rotational grazing and climate-based monitoring systems.

Due to a lack of knowledge or awareness regarding the life cycle of parasites in tropical climates, especially the important parasite Strongyles, or when the best time to use anthelmintics is, these drugs can become less effective over time, which makes them more expensive. The cost of anthelmintics for a single dose in the context of horse treatment often falls within the range of RM117 to RM186, as indicated by the prevailing prices offered by various anthelmintic suppliers in Malaysia. As a result, stable owners with more than 10 horses incur significantly higher expenses to treat the horses every three or six months in a year.

This article delves into the free-living stage of strongyles in a tropical setting, using Malaysia as a representative model. The country is recognised for its consistently high temperatures and diverse soil composition. A deeper understanding of the strongyle life cycle under these conditions is expected to improve parasite management in the tropics. It leads to more effective and efficient eradication strategies at farms, and the integration of environmental data with parasite surveillance can significantly improve the precision and sustainability of parasite management strategies (Morgan *et al*. 2023).

## MATERIALS AND METHODS

### Ethical Statement

No animal handling or discomfort occurred during sample collection for this study. Therefore, this study did not require ethical approval.

### Experimental Animals

Five adult horses aged between 22 and 24 years were selected. The horses were kept under a semi-intensive management system and had no history of recent anthelmintic treatment for the past six months.

### Experimental Design

This study employed a fully randomised design consisting of 16 experimental groups, each representing a distinct combination of environmental exposure:

1. An air-conditioned room with a regulated temperature of 21 ± 1°C.
2. A room with room temperature of 26 ± 1°C.
3. Outside the building under shelter that regulated mean temperature of 29 ± 1°C.
4. A room heated with a lamp that regulates the temperature at 32 ± 1°C and soil type (peat, clay, residual and sandy).

Each group included 30 experimental units, resulting in a total of 480 units. An experimental unit was defined as a 5 g soil sample that contained strongyle eggs derived from the faeces of naturally infected horses. Faecal samples, mixed with soil samples, were collected from naturally infected horses maintained at a stable, which had not received anthelmintic treatments for at least six months before sampling. This is to ensure the presence of viable strongyle eggs without confounding effects from recent drug administration.

While no replication was used at the group level, five subsamples were collected per unit, and larval counts were calculated as the mean of the five observations. The total number of experimental units (*N* = 480) was determined based on logistical feasibility and past studies, which have demonstrated that sample sizes of this scale are adequate to detect significant differences in larval development and survival across various soil and environmental conditions. The number of units per group (*n* = 30) was selected to allow sufficient statistical comparison across treatment groups.

The independent variables in this study were environmental exposure and soil type, as stated previously. The dependent variable was the number of third-stage larvae (L3) survivability from each soil unit. This observation was made over 30 consecutive days. Larval counts were counted per gram of soil and expressed as the mean of five subsamples per unit. Temperature and humidity at each site were continuously monitored using hygrometer loggers to maintain the parameters (Rose Vineer *et al*. 2020b).

### Parasitological Examinations

#### Faecal floatation and morphological identification

The horse’s fresh faeces were collected in the morning after their first meal. The faecal samples from each horse were individually analysed for strongyle egg count per gram (EPG) of faeces as a routine to confirm the presence of the strongyle eggs. Faecal floatation was carried out to detect strongyle-type eggs before culture.

**FIGURE 1:**
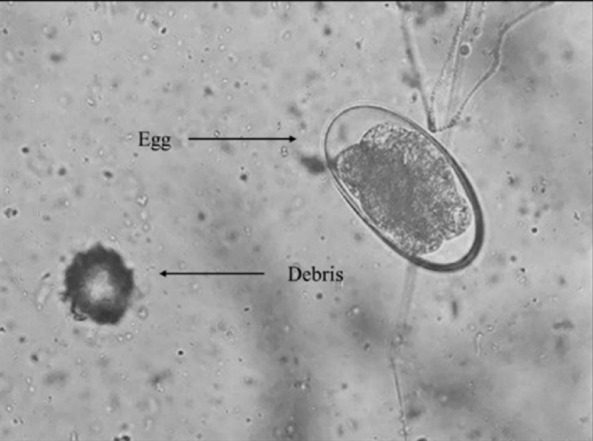
Strongyle eggs are oval, dark in the middle and lighter around the edge.

Four grams of faeces from the sample were mixed with 26 mL of saturated glucose solution (D-(+)-Glucose 5% w/v Solution, Chemiz) and stirred until well mixed. Then, the mixture was strained by using a strainer to separate the solution from the large residue and poured into a centrifuge tube. The centrifuge tube was then centrifuged at 165 rpm for 5 min. After that, the centrifuge tube was filled with a glucose-saturated solution until a meniscus formed on top of the tube. A coverslip was put on the meniscus to allow the eggs to float to the top of the centrifuge tube for 5 min. Then, the coverslip was placed on the slide, and the identification of the strongyle egg was performed using a light microscope at 40× and 100× magnification, confirming the morphology of the eggs, which are oval-shaped, with a lighter colour around the edges and darker in the middle (Fig. 1). Due to morphological similarity, genus identification was not possible via egg morphology (Bellaw & Nielsen 2015).

#### McMaster faecal egg count (FEC)

Quantification of strongyle egg burden was performed using the McMaster technique. Four grams of faeces from the sample were mixed with 26 mL of saturated glucose solution (D-(+)-Glucose 5% w/v Solution, Chemiz) and stirred until well mixed. Then, the mixture was strained by using a strainer to separate the solution from the large residue. The suspension was immediately loaded into both chambers of a McMaster slide (Eggzamin™) and left for 5 min. Under a light microscope with 40× and 100× magnification, the number of eggs present in the grids within both chambers of the slide was counted. By using the multiplication factor of 20 EPG, the total number of eggs presented in both chambers is multiplied by 20 to get the number of EPG (Vadlejch *et al*. 2011). This method is widely accepted for its simplicity and moderate sensitivity in estimating parasite loads.

#### Faecal culture and strongyle recovery (Baermann) technique

Faecal culture was used to develop strongyle-type eggs into their infective third-stage larvae (L3) under semi-natural, controlled conditions. In this study, faecal cultures were prepared by thoroughly mixing 1 kg of fresh feces with 1 kg of soils (peat, clay, residual and sandy) then placed in a wooden boxes and were incubated at four controlled different temperatures: 21 ± 1°C, 26 ± 1°C, 29 ± 1°C and 32 ± 1°C. During this time, the eggs hatched and progressed through the L1 and L2 stages before developing into the infective L3 stage (van Wyk & Mayhew 2013). The temperature of the ambience and soil was monitored using a hygrometer to maintain the parameters. The moisture of the mixture was retained by spraying with approximately 50 mL of water daily. Ventilation was provided in the area to prevent anaerobic conditions (Zhou *et al*. 2019)

A Baermann technique was used to determine larvae count per gram (LPG). A 10 g portion of faecal culture was placed in the funnel and soaked with approximately 50 mL of warm water. The setup was allowed to stand undisturbed for 12 h to 24 h. Motile larvae migrated out of the faecal mass, moved downward through the water, and collected at the bottom of the funnel. A few drops of Lugol’s iodine solution were used to immobilise the larvae for morphological identification under a light microscope of 40× and 100× magnification.

The morphology of the L1 and L2 of the strongyle larvae was determined by the oesophagus stages (Lyons & Tolliver 2015). Both L1 and L2 have a rhabditiform oesophagus, which has an anterior thick region and a posterior bulb. Meanwhile, L3 was determined by the number and shape of intestinal cells (IC) (Amer *et al*. 2022). The ratio of eggs and larvae, as well as the development and survivability of larvae, were observed for 30 consecutive days.

### Soil Analysis

The types of soil studied in this research are those commonly found in horse establishments in Malaysia. The four soils are peat, clay, residual and sandy soil. These soils are commonly found in the grazing paddocks and riding arenas. In this study, the soil samples were taken from identified places free from horses.

#### Physical characteristics

Samples of each soil type were analysed to determine the parameters of physical properties, including the soil’s texture and moisture (Food and Agriculture Organization of The United Nations 2020). The soil texture triangle is used to classify soil texture. The ribbon method was used to determine the moisture content in soil.

#### Chemical characteristics

The soil pH was assessed using an electrometric apparatus with a glass electrode sensitive to hydrogen ions (H^+^). Meanwhile, the salinity of the soil measuring nitrogen, phosphorus, cations potassium (K^+^), magnesium (Mg^2+^) and calcium (Ca^2+^), boron and ferum was measured using electrical conductivity (EC).

### Statistical Analysis

The statistical analysis was conducted using IBM SPSS Statistics (Version 26) to analyse the correlations (ANOVA) between different temperatures, types of soils and the development and survival rates of larvae. Differences between variables were considered significant at *p* < 0.05 for all statistical tests.

## RESULTS

The results elucidate the percentage of strongyle eggs, L1, L2 and L3 development over 30 days in four types of soil at four different environmental temperatures. The survival of L1, L2 and L3 cultured in four different types of soil and at various temperatures was observed over 30 days.

### Development Time of Strongyle Larval Stages

**FIGURE 2:**
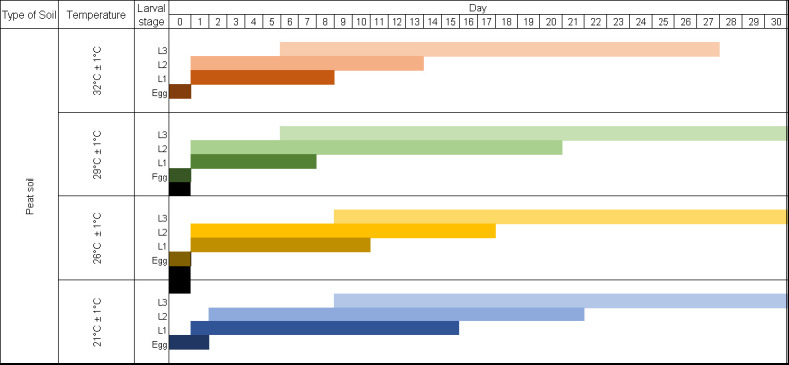

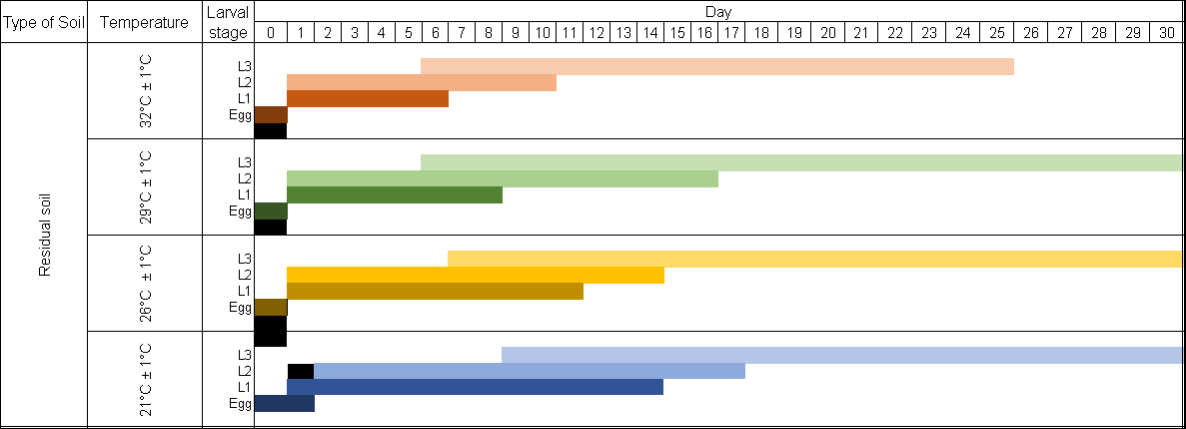

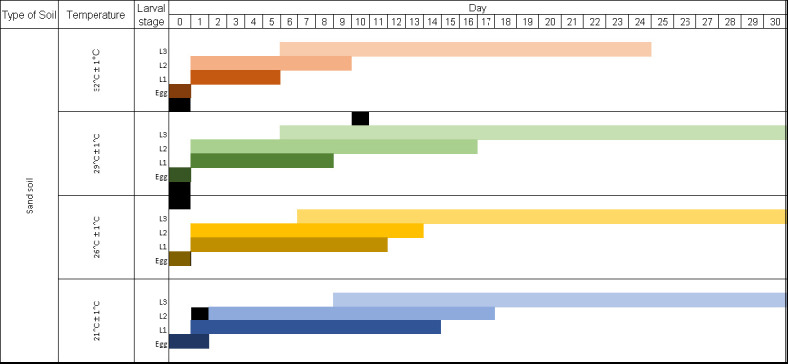

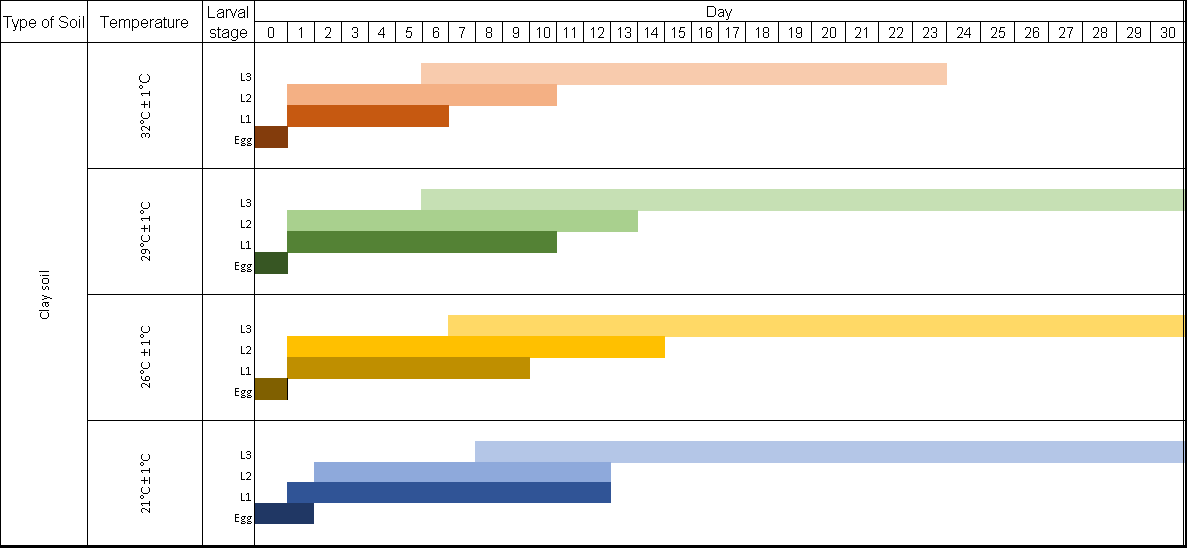
Time taken (day) by strongyle eggs to develop into L1, L2 and L3 in 21 ± 1°C, 26 ± 1°C, 29 ± 1°C and 32 ± 1°C environment temperatures on (a) peat soil; (b) residual soil; (c) sand soil; and (d) clay soil.

Figs. 2a, 2b, 2c and 2d each show the duration of strongyle eggs developed to free-living L1 and L3 in four different temperatures ranging from 21 ± 1°C to 32 ± 1°C on the peat, residual, sandy and clay soil, respectively. Across all soils (peat, residual, sandy and clay), progression to L1 occurred in 1 to 2 days, depending on the temperature; at 26°C–32°C, hatching was completed in 1 day, while at 21°C, it required 2 days. Development to L3 followed a clear temperature gradient: at 26°C, L3 appeared in 7–9 days; at 29°C–32°C, this period shortened to 5–6 days (Figs. 2a–d).

**FIGURE 3:**
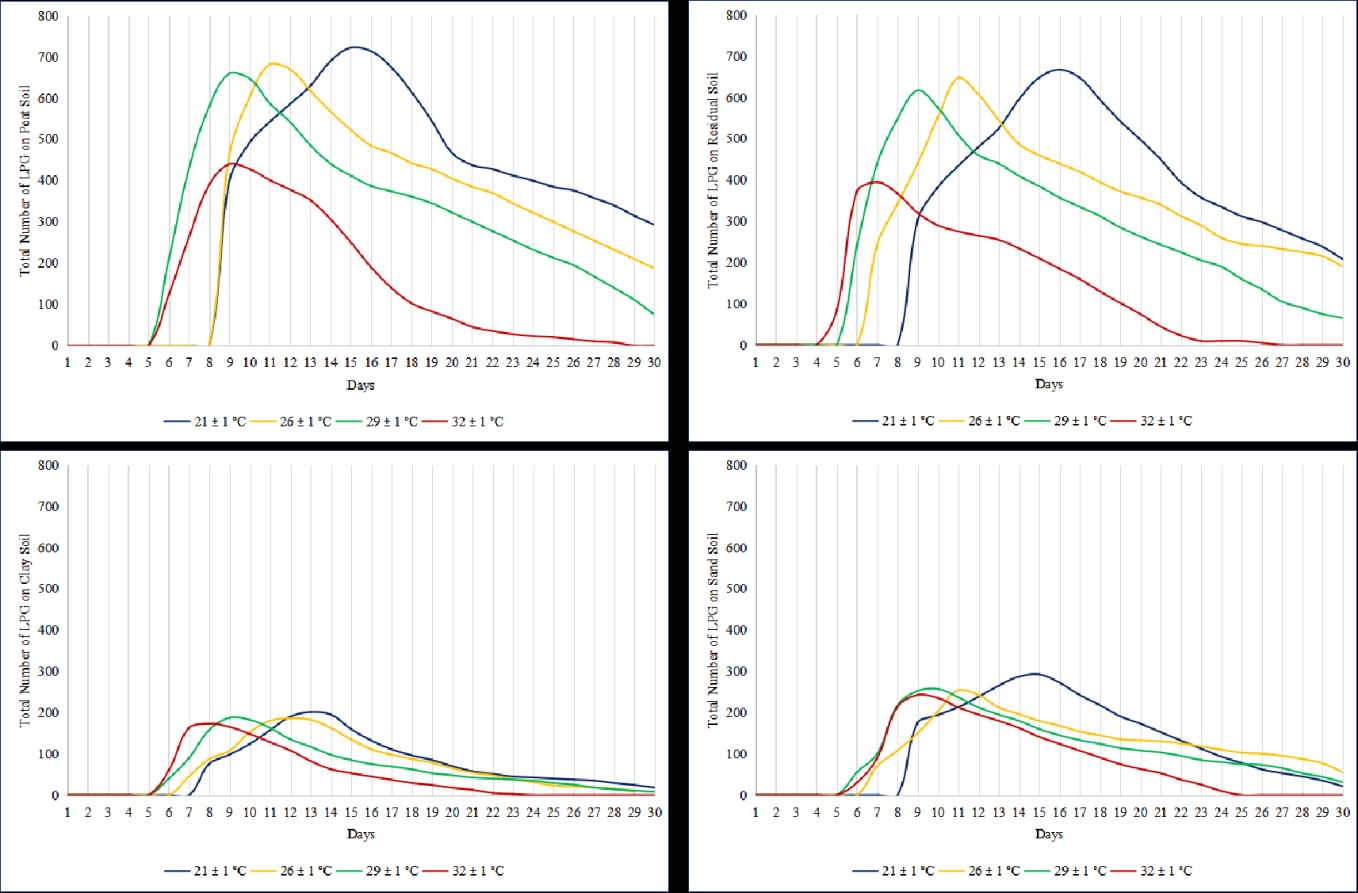
Development of L3 at different temperatures on four soil types.

The survival of L3 was observed at four different temperatures and in four different soils for 30 days. The L3 survived all four types of soils throughout the observation period at temperatures 21 ± 1°C, 26 ± 1°C, and 29 ± 1°C. However, at a temperature of 32 ± 1°C, L3 only survived up to D27, which varies depending on the type of soil. Fig. 3 shows that L3 survived for up to 27 days on peat soil, 25 days on residual soil and 24 days on sandy soil, respectively, and for 23 days on clay soil.

There was a significant difference between the temperatures and time taken for the development of strongyle larvae from eggs into L3 (*p* < 0.05). There was no significant difference in the development of larvae in all four soil types.

### Survivability of L3 in Different Temperatures and Soil Types

L3 survival declined with increasing temperature across soils. At 21°C, survival to Day 30 ranged from 41% (clay) to 68% (peat); at 26°C, 35%–58%; at 29°C, 34%–55%; and 32°C, survival dropped sharply (21%–45%) with larvae surviving only until Days 23–27 (Table 1). Survival differences across soil types and temperatures were significant (*p*< 0.05).

## DISCUSSION

This study tested the hypothesis that warmer temperatures would accelerate strongyle larval development but shorten their survivability, while soil type would influence larval survival but not development duration. The objectives were:

1. To quantify the development and survival of strongyle larvae in peat, residual, sandy and clay soils across four temperature regimes.
2. To inform pasture management strategies for grazing horses.

The results confirmed that temperature significantly influences larval development, with L3 larvae appearing in 9 days at 26°C and accelerating to 5–6 days at 29°C–32°C. These findings align with those of Merlin *et al*. (2022), who documented faster development at higher temperatures, and complement earlier laboratory studies (Mfitilodze & Hutchinson 1987; Nielsen *et al*. 2007), which reported optimal development between 20°C and 33°C and accelerated larval maturation at approximately 28°C. However, survival decreased sharply at high temperatures: L3 persisted only until Days 23–27 at 32°C, consistent with studies by Nielsen *et al*. (2007), which showed reduced larval longevity above 30°C.

While soil type influenced absolute survival percentages, it did not affect development timing. Peat soil exhibited the highest L3 survival (68% at 21°C) due to its high moisture retention, whereas clay and sandy soils showed lower survival rates. Kuzmina *et al*. (2006) demonstrated prolonged larval persistence in damp soils, and Salley *et al*. (2018) and Pepper and Brusseau (2019b) further emphasised the role of soil texture and moisture in larval survival, indicating that soil influences larval persistence mainly through moisture retention rather than developmental inhibition.

The results of this study show that the larvae took a longer time to develop but survived for more than 30 days on peat soil at relatively cooler temperatures in a tropical environment. Unlikely on L3 on clay soil, they developed more quickly but survived for a shorter period. On a global scale, the larvae took more time to mature. In temperate countries, the larvae took 33 to 48 days to mature to the L3 stage in cold temperatures (7.5°C to 8°C), whereas the time was significantly shorter (1.7 days) at 37°C (Merlin *et al*. 2022).

Peat soil, with higher moisture retention, provided the longest L3 survivability (up to 68% at 21°C), highlighting its role as a reservoir of refugia. This observation is supported by field studies demonstrating enhanced larval persistence in moist substrates (Kuzmina *et al*. 2006). Clay and sandy soils exhibited lower survival rates under comparable conditions, indicating that bulk density and moisture constraints negatively impact larval viability. Therefore, peat soil’s moisture retention and high organic matter content likely contributed to higher larval survivability, in contrast to clay and sandy soils (Salley *et al*. 2018; Pepper & Brusseau 2019a). Naturally, clay soil is heavy and dry, but peat soil has a loamy texture and is wet (Salley *et al*. 2018).

A study reveals that moisture, or humid soil, influences the survival of L3 larvae and the development of the infective stage (Purwati *et al*. 2017). Pores of water-saturated soils are filled with water, while dry soils have a relatively low soil moisture content because most of the pores in the soil are filled with air (Pepper & Brusseau 2019b). Studies by Blazejak *et al*. (2022) and research on pasture rewetting consistently link elevated soil moisture to higher parasite infection risk, supporting our observation that peat’s high moisture content fosters L3 refugia. Weiss *et al*. (1998) modelled peat soil moisture retention, reinforcing peat’s role as a larval survival medium. Peat soil can retain moisture more effectively, which increases the survival rate of larvae compared to that on clay soil.

The application rates of nitrogen and phosphorus fertilisers influenced the soil nematodes. High nitrogen levels in the soil can inhibit the free-living stage of parasitic larvae, while low phosphorus levels may hinder their development (Ni *et al*. 2024). However, in this study, the soil abundance of nitrogen had a higher number of larvae that survived. This finding contradicts the earlier study, and further research is warranted to investigate this discrepancy. In addition to that, the soil contains low amounts of exchangeable cations such as potassium (K^+^), magnesium (Mg^2+^), and calcium (Ca^2+^), and high ferum creates favourable conditions for parasites to thrive. A study revealed that exchangeable cations have a positive impact on parasites (Sumboh *et al*. 2023).

To conclude, parasites tend to thrive in neutral and mildly acidic environments, as opposed to those with higher acidity and alkalinity levels. Therefore, the combination of abundant nitrogen, exchangeable cations and optimal pH levels could create the perfect conditions for larvae to grow and multiply. The texture and moisture also contribute to the high larva counts as the soil is loam and has a moist texture. These findings shed light on the complex interactions between soil nutrients and parasite development, highlighting the need for further research in this area.

The interplay of temperature, soil, and moisture has been well-documented globally. English (1979), Hutchinson *et al*. (1989) and Ogbourne (1973) all emphasised the key role of moisture in larval migration in tropical climates and temperature in temperate zones. Moreover, Kuzmina *et al*. (2006) reported that overwintering L3 larvae occurred under faecal pats, and Osterman-Lind *et al*. (2022) found that strongyle larvae survived up to 3 months at 20°C–28°C in faeces. These findings align with our observations from tropical temperatures and support the role of peat as a refuge.

These findings have direct relevance to equine parasite control. In tropical climates, rapid L3 development at higher temperatures increases infection pressure but may be offset by decreased survival, limiting long-term pasture contamination. Nonetheless, peat-based or shaded paddocks should be avoided during wet and cool seasons, as they may sustain L3 refugia for more than 30 days, thereby extending the risk of infection. On clay-based soils during heat, L3 may persist for only ~23 days, reducing the infectivity period.

The hypothesis for this study is supported: temperature significantly affects both the duration of development and the survivability of larvae, but soil type only impacts survival, not development. Future investigations, such as field trials (e.g., pasture paddock rotation studies), are needed to validate these findings and explore the role of faecal moisture and microbial interactions in L3 persistence.

## CONCLUSIONS AND SUGGESTIONS

Peat soil is conducive for the development of strongyle eggs to the L3 stage, with the highest count observed on Day 9 at 29 ± 1°C. Sandy and clay soils are detrimental to the larvae, as evidenced by a low percentage of L3 larvae that developed and did not survive beyond 25 days at 32 ± 1°C (*p* < 0.05). Paddocks with peat soil can be used for grazing for up to 6 days during the cool, wet season and 5 days during the warm season, and then rotated for 30 days to ensure effective parasite management.

From this study, pasture management is one of the methods to eradicate parasites in the field. This includes scheduled rotational grazing of determined areas to reduce L3 exposure to animals. It is recommended not to use peat soil paddock for horses to graze during wet and cool temperatures because the survival rate of L3 lasts for more than 30 days at the highest count among the studied soil types.

## Figures and Tables

**TABLE 1:** The parameters of soil and the survivability rate of larvae among the different temperatures and soils.

Parameter of soil	Peat soil	Residual soil	Sandy soil	Clay soil	*p*-value
Total nitrogen (%)	1.17	0.24	0.07	0.03	< 0.05
Total phosphorus (Mg/kg)	982	171	235	45	0.231
Exchangeable cation K^+^ (Me/kg)	0.05	0.11	0.05	0.12	< 0.05
Exchangeable cation Mg^2+^ (Me/kg)	0.04	0.16	0.07	0.15	< 0.05
Exchangeable cation Ca^2+^ (Me/kg)	0.44	0.7	0.4	4.2	< 0.05
Boron (Me/kg)	< 0.1	< 0.1	16	< 0.1	0.446
Ferum (%)	2.67	2.53	0.11	1.95	< 0.05
pH value	7	6.5	6	8	< 0.05
Texture	Loam	Sandy clay	Sandy loam	Clay	
Moisture (%) at 21 ± 1°C	75–100	75–100	50–75	50–75	
Moisture (%) at 26 ± 1°C	50–75	50–75	50–75	50–75	
Moisture (%) at 29 ± 1°C	50–75	50–75	50–75	50–75	
Moisture (%) at 32 ± 1°C	25–50	25–50	0–25	0–25	
L3 survival rate (%) at 21 ± 1°C	68	63	50	41	< 0.05
L3 survival rate (%) at 26 ± 1°C	58	56	44	35	< 0.05
L3 survival rate (%) at 29 ± 1°C	55	51	45	34	< 0.05
L3 survival rate (%) at 32 ± 1°C	45	38	36	21	0.594
p-value	< 0.05	< 0.05	< 0.05	< 0.05	

Notes: K = potassium; Mg = magnesium; Ca = calcium
